# Amplification of ACK1 promotes gastric tumorigenesis via ECD-dependent p53 ubiquitination degradation

**DOI:** 10.18632/oncotarget.6194

**Published:** 2015-10-20

**Authors:** Song-Hui Xu, Jin-Zhou Huang, Min Chen, Ming Zeng, Fei-Yan Zou, De Chen, Guang-Rong Yan

**Affiliations:** ^1^ Institutes of Life and Health Engineering, Jinan University, Guangzhou, China; ^2^ Biomedicine Research Center and Department of Surgery, The Third Affiliated Hospital of Guangzhou Medicine University, Guangzhou, China; ^3^ Key Laboratory for Major Obstetric Diseases of Guangdong Province, Key Laboratory of Reproduction and Genetics of Guangdong Higher Education Institutes, Guangzhou, China

**Keywords:** ACK1, gastric cancer, amplification, p53

## Abstract

Amplification or over-expression of an activated Cdc42-associated kinase 1 (ACK1) gene is common in breast, lung and ovarian cancers. However, little is known about the role of ACK1 in gastric tumorigenesis. Here, we found that DNA copy numbers of the ACK1 gene and its mRNA expression levels were significantly increased in gastric cancer (GC) compared to normal gastric tissues. Additionally, silencing ACK1 inhibited GC cell proliferation and colony formation, induced G2/M arrest and cellular apoptosis *in vitro*, and suppressed tumor growth *in vivo*. Gene Ontology annotation revealed that 147 differential proteins regulated by ACK1 knockdown were closely related with cellular survival. A cell cycle regulator, ecdysoneless homolog (ECD), was found to be significantly down-regulated by ACK1 knockdown. Silencing of ECD inhibited colony formation and induced G2/M arrest and cell apoptosis, which is similar to the effects of ACK1 knockdown. Silencing of ECD did not further enhance the effects of ACK1 knockdown on G2/M arrest and apoptosis, while silencing of ECD blocked the enhancement of colony formation by ACK1 over-expression. Over-expression of ACK or ECD promoted the ubiquitination of tumor suppressor p53 protein and decreased p53 levels, while silencing of ACK1 or ECD decreased the p53 ubiquitination level and increased p53 levels. Silencing of ECD attenuated the ubiquitination enhancement of p53 induced by ACK1 over-expression. Collectively, we demonstrate that amplification of ACK1 promotes gastric tumorigenesis by inducing an ECD-dependent ubiquitination degradation of p53.

## INTRODUCTION

Gastric cancer (GC) is one of the most common malignancies in the world, and it has poor prognosis and limited treatment options, accounting for 10% of all cancer-related deaths and 8% of all cancer cases [1]. More than 90% of GCs are gastric adenocarcinomas. Although surgical resection is still considered the gold standard for treating GC patients, the prognosis of patients with GC remains poor due to the high incidence of tumor recurrence and distant metastasis, as most patients with GC do not respond to current chemotherapies or radiotherapies [2]. Targeted small molecule or antibody therapies designed to inhibit a specific addictive oncogene are a promising therapeutic strategy [3]. Therefore, there is an urgent need for further understanding of the molecular mechanisms in GC tumorigenesis and for the discovery of new therapeutic targets.

It has been established that gastric tumorigenesis is caused by a complex interaction between the host and environmental factors [4]. Copy number variation refers to a form of genomic structural variation that results in abnormal gene copy numbers, including gene amplification, gain, loss and deletion. The gene amplification of activated Cdc42-associated kinase 1 (ACK1) is common in breast, lung and ovarian cancers [5–8]. ACK1 was originally identified as a non-receptor tyrosine kinase that bound to the GTP-Cdc42 complex and inhibited its GTPase activity [9]. Over-expression of ACK1 is associated with cancer progression and metastasis in breast cancer, prostate cancer, non-small-cell lung cancer and hepatocellular carcinoma [5, 10–15]. Our previous study showed that ACK1 is significantly up-regulated in GC compared to adjacent gastric tissues and is associated with a poor prognosis in GC patients [16]. To date, however, the impact of ACK1 on GC tumorigenesis and its molecular mechanisms in GC have not been elucidated.

In this study, we demonstrate that ACK1 DNA copy number and mRNA levels are significantly higher in GC compared to normal gastric tissue. The silencing of ACK1 inhibited cell proliferation and colony formation, induced G2/M arrest and apoptosis *in vitro*, and suppressed tumor growth *in vivo*. Mechanistically, ACK1 induced p53 ubiquitination degradation and decreased levels of p53, a key cell cycle and apoptosis regulator, by up-regulating ECD. Herein, we elucidate a novel molecular mechanism by which ACK1 stimulates gastric tumorigenesis.

## RESULTS

### Amplification and over-expression of the ACK1 gene in GC tissues

**Figure 1:**
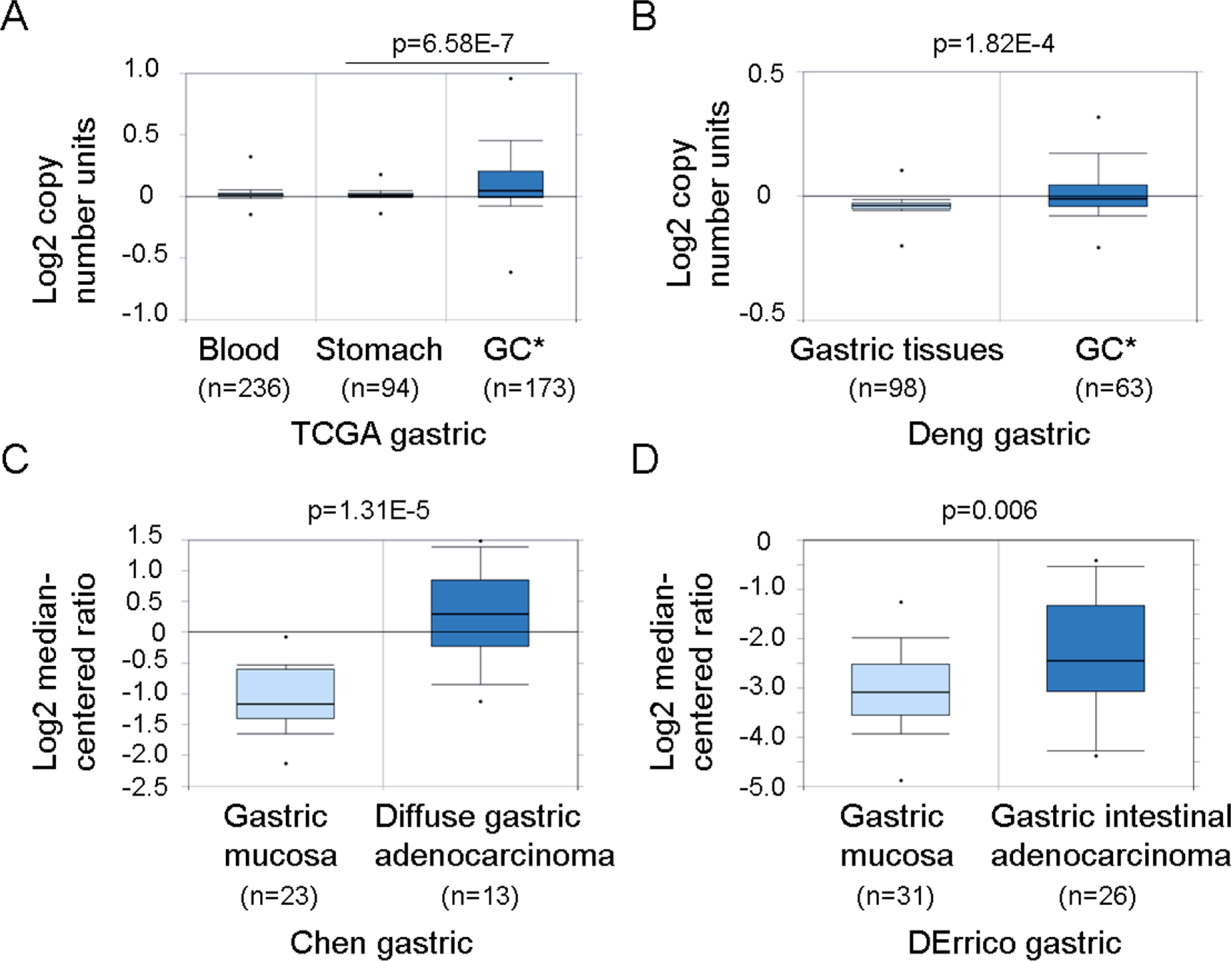
Amplification and over-expression of the ACK1 gene in gastric carcinoma **(A)** DNA copy numbers of the ACK1 gene were increased in gastric adenocarcinoma (GC*) compared to normal stomach tissues and blood (whole blood genomics DNA) by analyzing TCGA gastric database from Oncomine. **(B)** DNA copy numbers of the ACK1 gene were increased in GC* compared to normal gastric tissues when analyzing the Deng gastric database from Oncomine. **(C)** ACK1 mRNA levels were up-regulated in the diffuse gastric adenocarcinoma compared to gastric mucosa by analyzing the Chen gastric database from Oncomine. **(D)** ACK1 mRNA levels were increased in gastric intestinal adenocarcinoma compared to gastric mucosa in the Derrico gastric database.

Our previous study showed that ACK1 protein levels and ACK1 phosphorylation at Tyr 284 were frequently elevated in GC and associated with poor survival in GC patients [16]. Amplification of the ACK1 gene is frequent in breast, lung and ovarian cancers [6, 8]. FISH analyses using 10 bacterial artificial chromosome probes containing loci encoding 10 protein kinases suggested that the ACK1 gene may be amplified in GC tissues [17]. To further investigate whether the ACK1 gene is amplified in GC tissues, ACK1 DNA copy numbers in gastric adenocarcinoma and normal gastric tissues were analyzed using two DNA datasets deposited in the Oncomine database (https://www.oncomine.org/resource/login.html). Significantly higher ACK1 DNA copy numbers were observed in gastric adenocarcinoma compared to normal stomach tissues and whole blood in the TCGA gastric dataset (*p* = 6.58E-7) (Figure 1A). Similarly, higher ACK1 DNA copy numbers were also noted in gastric adenocarcinoma compared to gastric tissues in the Deng gastric dataset (*p* = 1.82E-4) (Figure 1B) [18]. These observations indicate that the ACK1 gene is amplified in gastric carcinoma.

The mRNA levels of ACK1 between normal gastric tissues and GC tissues were further investigated using two microarray gene expression datasets deposited in the Oncomine database. Higher ACK1 mRNA levels were observed in diffuse gastric adenocarcinoma or gastric intestinal adenocarcinoma compared to gastric mucosa tissues in the Chen and Derrico gastric datasets, respectively (Figure 1C and 1D) [19, 20], suggesting that ACK1 expression was up-regulated in GC. All of these findings in different independent datasets indicate that the ACK1 gene is amplified and its expression is increased in GC, suggesting that ACK1 may play an important role in gastric tumorigenesis.

### Silencing of ACK1 inhibits tumor growth *in vitro* and *in vivo*

**Figure 2:**
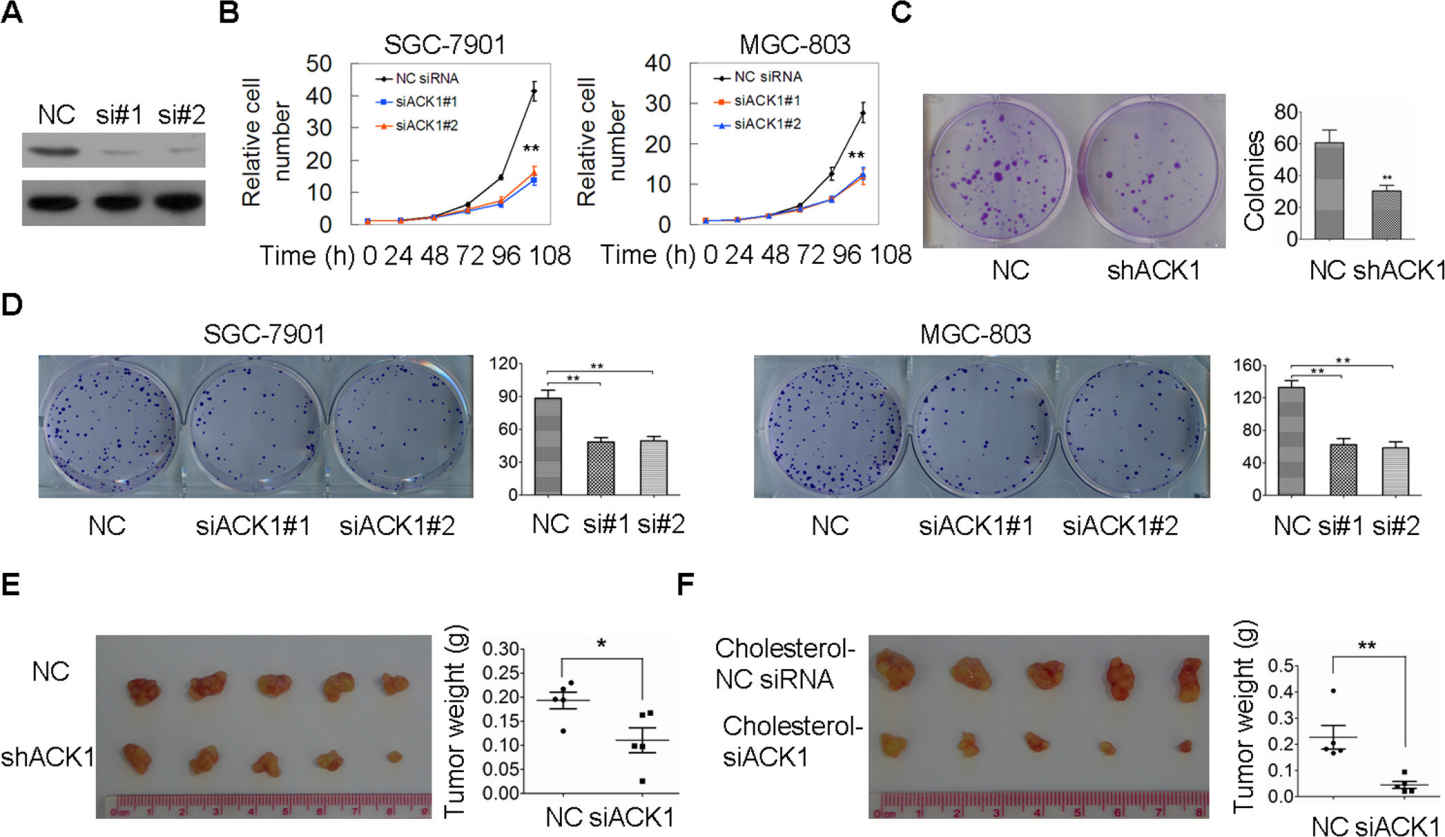
Silencing of ACK1 inhibits cell proliferation and colony formation *in vitro* and tumor growth *in vivo* **(A)** SGC-7901 cells were transfected with the indicated anti-ACK1 siRNAs for 48 h. ACK1 protein level was determined by western blotting. **(B)** SGC-7901 and MGC-803 cells were transfected with the indicated siRNAs for the indicated time, and the cell number was counted. **(C)** Colony formation abilities between SGC-7901-ACK1 shRNA cells with stable ACK1 knockdown and SGC-7901-NC shRNA cells were measured for two weeks. The image and colony numbers of cellular clones with more than 30 cells are shown in the left and right sides (*n* = 3). **(D)** SGC-7901 and MGC-803 cells were transfected with the indicated anti-ACK1 siRNAs, colony formation abilities of these cells were measured after two weeks (*n* = 3). **(E)** The in vivo growth of the indicated cell lines with stable ACK1 knockdown were examined as described in the Materials and Methods. The images and weight of xenograft tumors are shown in the left and right panel, respectively (*n* = 5). **(F)** The xenograft tumor mouse model were intratumorally injected with cholesterol-conjugated siACK1 or NC siRNAs, the images and weight of xenograft tumors are shown in the left and right panel, respectively (*n* = 5).

To determine the functional roles of ACK1 in gastric tumorigenesis, ACK1 expression was silenced (Figure 2A, Supplementary Figure S1). We found that GC cell proliferation was obviously decreased when ACK1 was knocked down (Figure 2B). Silencing of ACK1 inhibited cell colony formation in gastric cancer cells SGC-7901 and MGC-803 (Figure 2D). Additionally, a stable transfectant of a specific shRNA targeted ACK1 in an SGC-7901 cell line was constructed. Cell colony formation was significantly inhibited when ACK1 expression was stably silenced (Figure 2C). More importantly, as shown in Figure 2E, the GC xenograft tumor growth of the stable transfectant was clearly impaired *in vivo* when ACK1 was knocked down in SGC-7901 GC cells. We further demonstrated that the intratumoral injection of cholesterol-conjugated siACK1 significantly inhibited gastric tumor growth (Figure 2F). Therefore, we concluded that ACK1 plays an essential role in GC cell proliferation, colony formation and tumor growth, indicating that ACK1 participates in GC tumorigenesis.

### Silencing of ACK1 induces G2/M arrest and cell apoptosis

**Figure 3:**
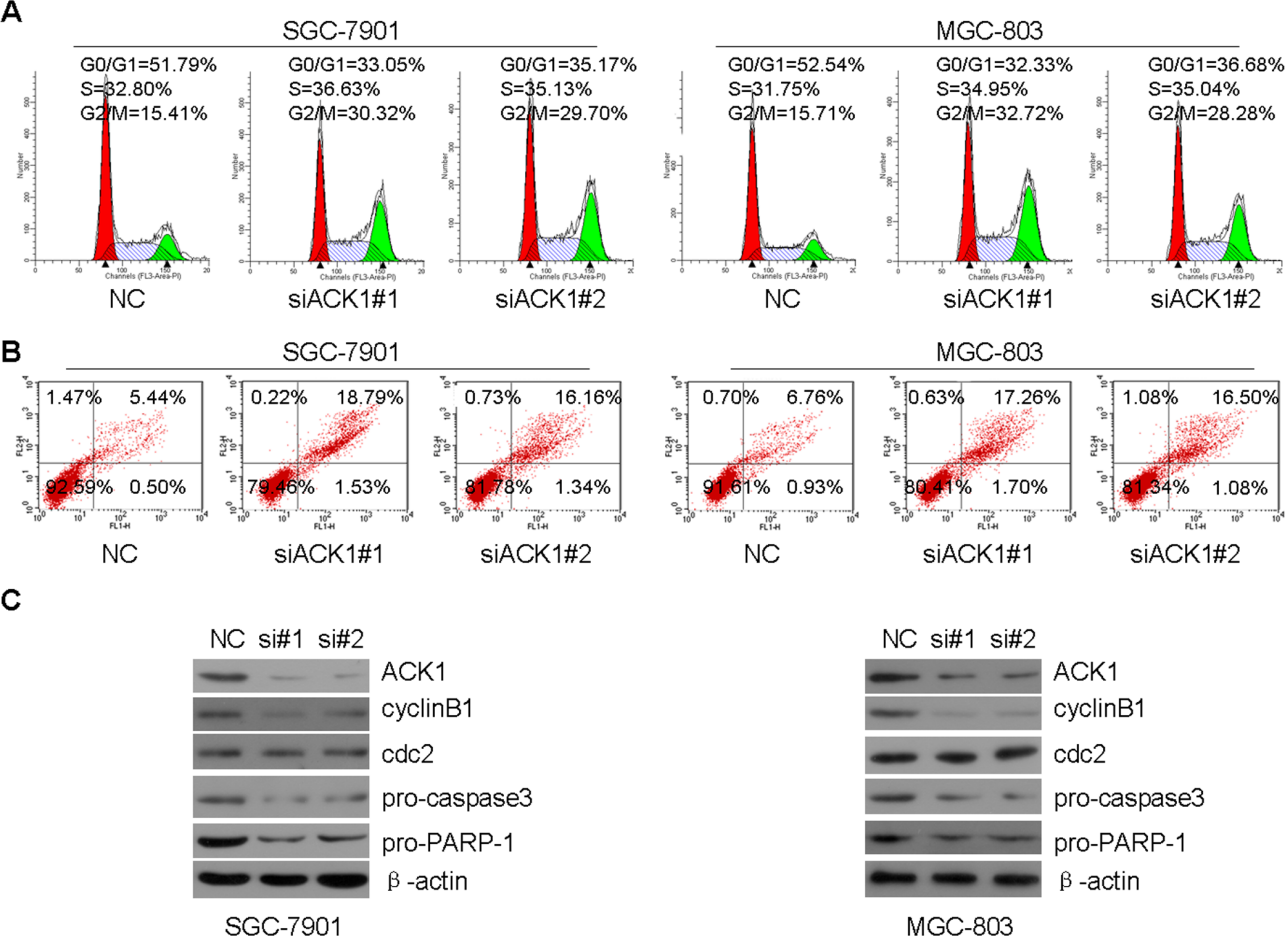
Knockdown of ACK1 induces G2/M arrest and cellular apoptosis in GC cells **(A)** SGC-7901 and MGC-803 cells were transfected with the indicated siRNAs for 48 h, the distribution of cell cycle was measured by flow cytometry. **(B)** SGC-7901 and MGC-803 cells were transfected with the indicated siRNAs for 72 h, and cellular apoptosis was determined by flow cytometry. **(C)** SGC-7901 and MGC-803 cells were transfected with the indicated siRNA for 48 h, and the indicated proteins were detected by western blot.

The dysregulation of cell cycle transition and cellular apoptosis are two important features of tumorigenesis. To explore how ACK1 silencing inhibited gastric tumor growth, the influences of ACK1 knockdown on cell cycle and apoptosis were further investigated using flow cytometry. When ACK1 in GC cells was silenced by siACK1#1 and siACK1#2 for 48 h, we found that ACK1 silencing induced GC cell G2/M arrest in SGC-7901 and MGC-803 GC cells (Figure 3A) and decreased the level of cyclin B, a key regulator of G2/M transition (Figure 3C). Cellular apoptosis is subsequently induced when cell arrest is not repaired. Cell apoptosis was obviously induced by ACK1 knockdown after 72 h in SGC-7901 and MGC-803 GC cells (Figure 3B), and the apoptosis markers pro-caspase3 and pro-PARP-1 were also decreased by ACK1 knockdown (Figure 3C). Together, these data indicate that silencing of ACK1 inhibits tumor growth by inducing G2/M arrest and apoptosis.

### ACK1-regulated proteins are associated with cellular survival

**Figure 4:**
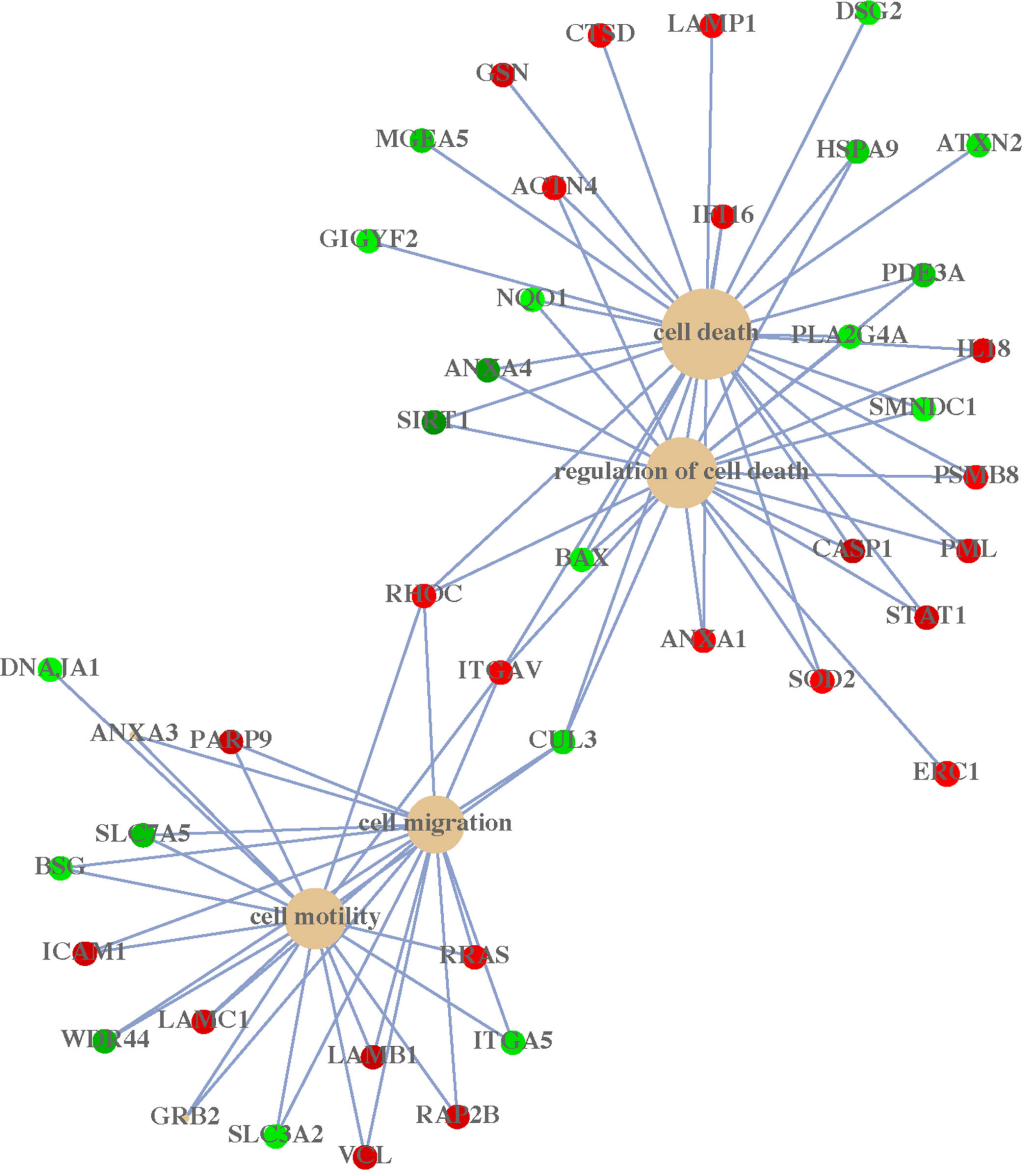
Concept-gene networks of enriched biological processes of ACK1-regulated proteins were analyzed by Gene Ontology annotation

To elucidate the molecular mechanism of ACK1 on the regulation of tumor growth and colony formation, 147 differential proteins regulated by ACK1 were previously identified using SILAC quantitative proteomics by our group [16]. Herein, a gene ontology annotation analysis further revealed that 147 differential proteins regulated by ACK1 could be categorized into two main groups (regulation of cell death (survival) and cell migration) according to their associated biological processes (Figure 4). Our previous study has reported the functional roles and molecular mechanism of ACK1 in cell migration [16]. Here, we mainly focused on the roles and molecular mechanism of ACK1 in cellular survival (anti-death). Many proteins associated with cell death were identified when ACK1 was knocked down, suggesting that the amplification of the ACK1 gene in GC cells is closely related to cancer cell survival, which is consistent with our observed effects.

### ACK1 promotes tumor growth by up-regulating ECD

**Figure 5:**
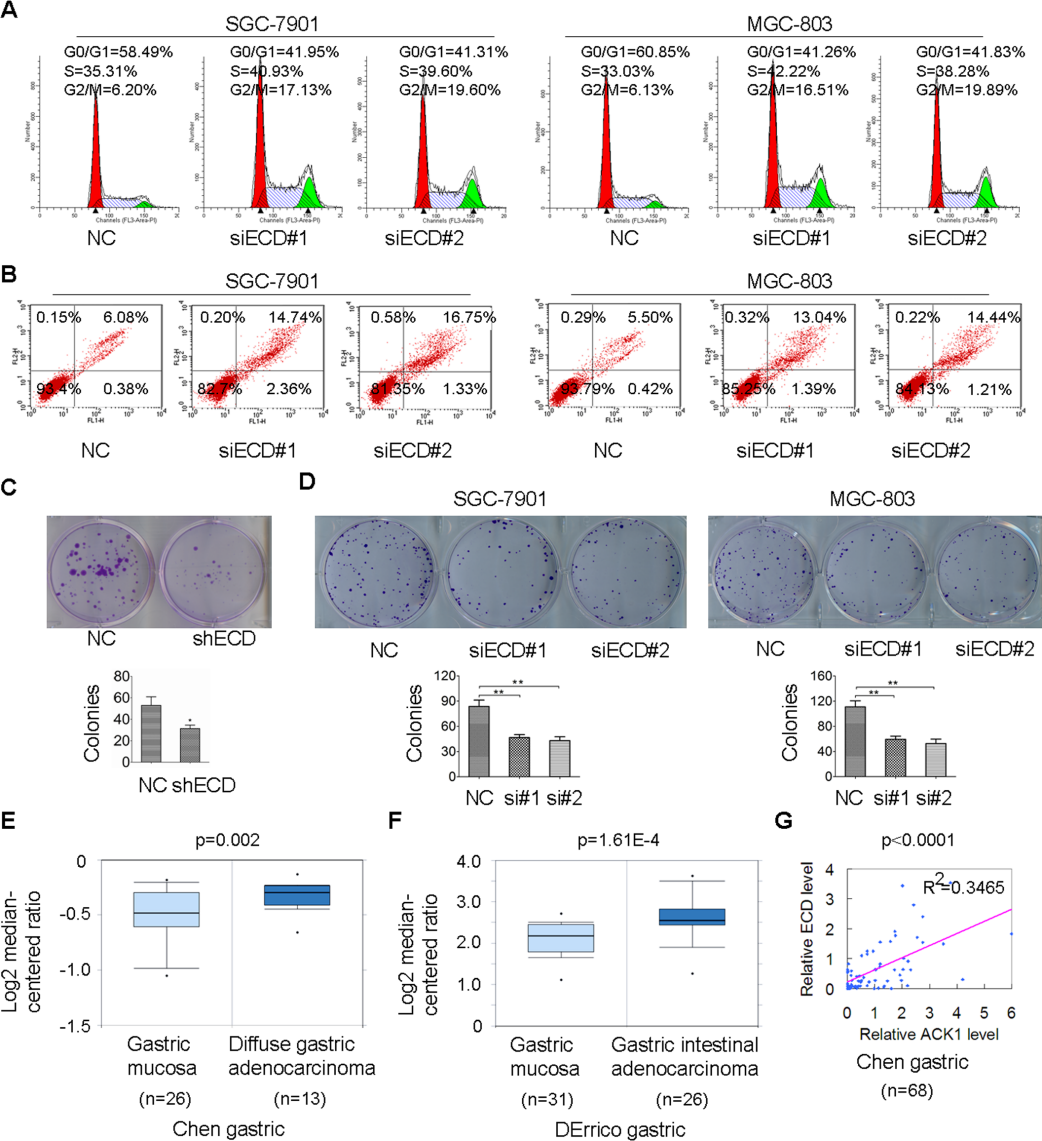
ECD mRNA levels are up-regulated in GC compared to normal gastric mucosa, and silencing of ECD induces G2/M arrest and apoptosis, and inhibits colony formation in GC cells **(A, B)** SGC-7901 and MGC-803 cells were transfected with the indicated siRNAs, the distribution of cell cycle **(A)** and apoptosis **(B)** were measured using flow cytometry. **(C)** Colony formation abilities between SGC-7901-ECD shRNA cells with stable ECD knockdown and SGC-7901-NC shRNA cells were measured for two weeks. The image and colony numbers of cellular clones with more than 30 cells are shown in the up and down sides (*n* = 3). **(D)** SGC-7901 and MGC-803 cells were transfected with the indicated siRNAs, colony formation abilities were detected after two weeks transfection. **(E)** ECD mRNA levels were up-regulated in the Figure 1C database. **(F)** ECD mRNA levels were increased in the Figure 1D database. **(G)** Analysis showing linear regressions and Pearson correlations of ACK1 with ECD in gastric tumor samples in the Figure 1C database (*n* = 68).

Among the 147 ACK1-regulated differential proteins, ECD (also known as hSGT1), is an interesting protein because of its marked decrease by ACK1 knockdown. The human ECD gene was first identified in a complementation assay conducted to rescue a growth defect in saccharomyces cerevisiae mutants with a GCR2 gene deletion [21]. An ECD deletion in murine embryonic fibroblasts resulted in G1/S arrest through the removal of competitive binding to the Rb protein and subsequent down-regulation of E2F target gene expression [22]. Recently, ECD over-expression has been closely associated with a poor prognosis in pancreatic cancer [23]. Our previous study showed that ACK1 promotes gastric cancer EMT and metastasis by AKT1-POU2F1-ECD signaling axis [16]. Whether ECD also plays an essential role for ACK1 in the regulation gastric cancer growth and survival is still unclear. Here, we demonstrated that silencing ECD induced GC cell G2/M arrest and apoptosis and inhibited GC cell proliferation and colony formation in SGC-7901 and MGC-803 GC cells, similar to the effects of ACK1 knockdown (Figure 5A–5D, Supplementary Figure S2). Furthermore, ECD expression levels were investigated in gastric carcinoma. Higher ECD mRNA levels were observed in diffuse gastric adenocarcinoma or gastric intestinal adenocarcinoma compared to gastric mucosa tissues in the Chen and Derrico gastric datasets [19, 20], respectively (Figure 5E and 5F). To determine whether ECD up-regulation is associated with ACK1 over-expression in gastric cancer samples, the correlation of ECD with ACK1 was further analyzed in Chen gastric dataset. Our results demonstrated that expression of ECD was significantly and positively correlated with ACK1 expression (*R*^2^ = 0.3465, *p* < 0.0001) (Figure 5G).

**Figure 6:**
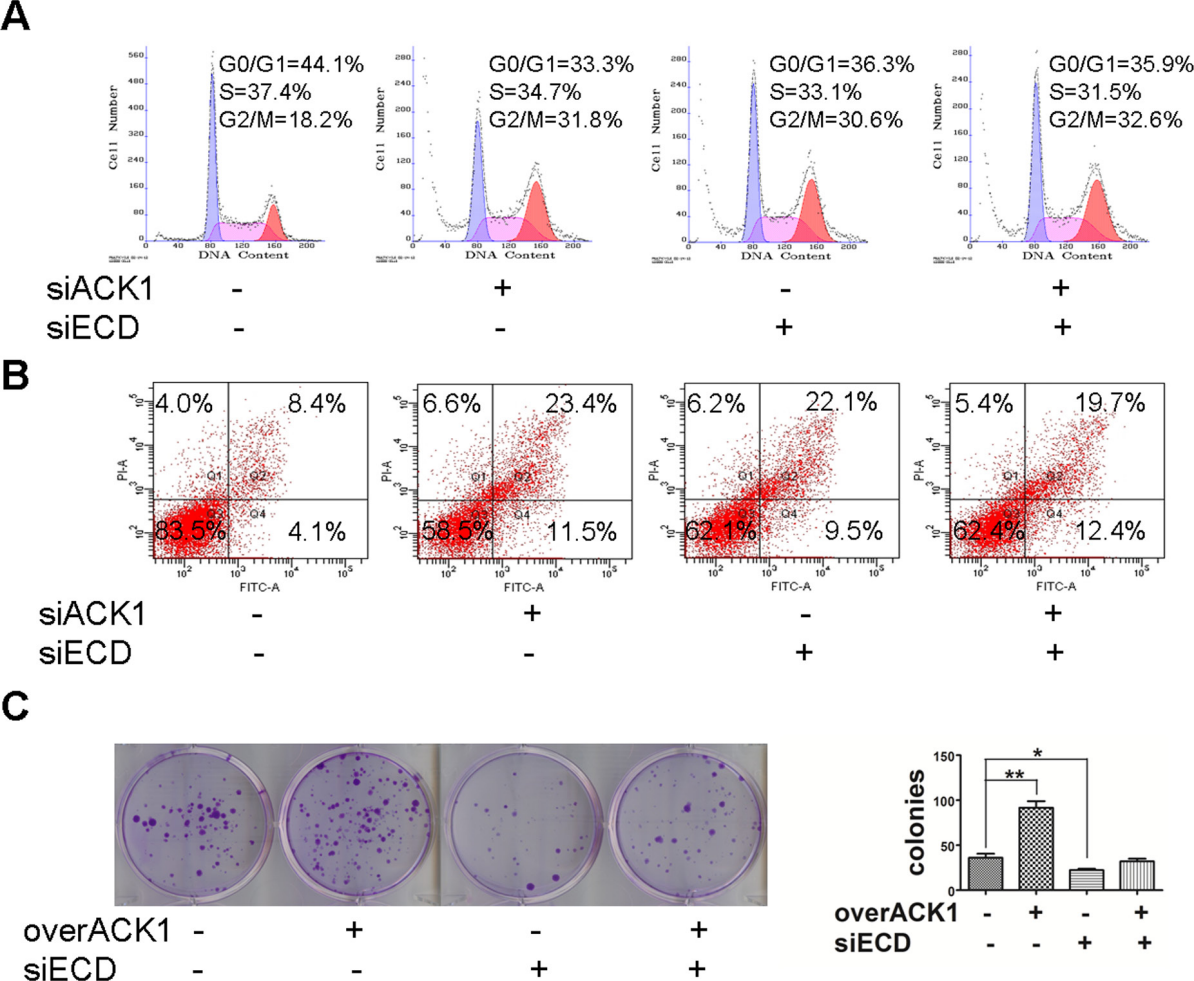
Silencing of ECD blocks the phenotypes generated by ACK1 over-expression **(A, B)** SGC-7901 cells were transfected with the indicated siRNAs, and the distribution of cell cycle **(A)** and apoptosis **(B)** were measured using flow cytometry. **(C)** SGC-7901 cells were transfected with an ACK1 plasmid together with anti-ECD siRNA, and colony formation abilities were measured for two weeks. The image and colony numbers of cellular clones with more than 30 cells are shown in the left and right sides (*n* = 3).

The silencing of ECD did not further enhance the effects of ACK1 knockdown on G2/M arrest and apoptosis because ECD expression has been significantly lower when ACK1 was silenced (Figure 6A and 6B), suggesting that ECD is an important downstream effector of ACK1 in the regulation of tumor growth and cellular survival. However, silencing of ECD blocked the enhancement of ACK1-induced colony formation (Figure 6C). Collectively, our data indicates that ACK1 promotes gastric cancer growth and survival mainly by up-regulating ECD expression.

### ACK1 promotes GC growth and survival by inducing ECD-dependent p53 ubiquitination degradation

**Figure 7:**
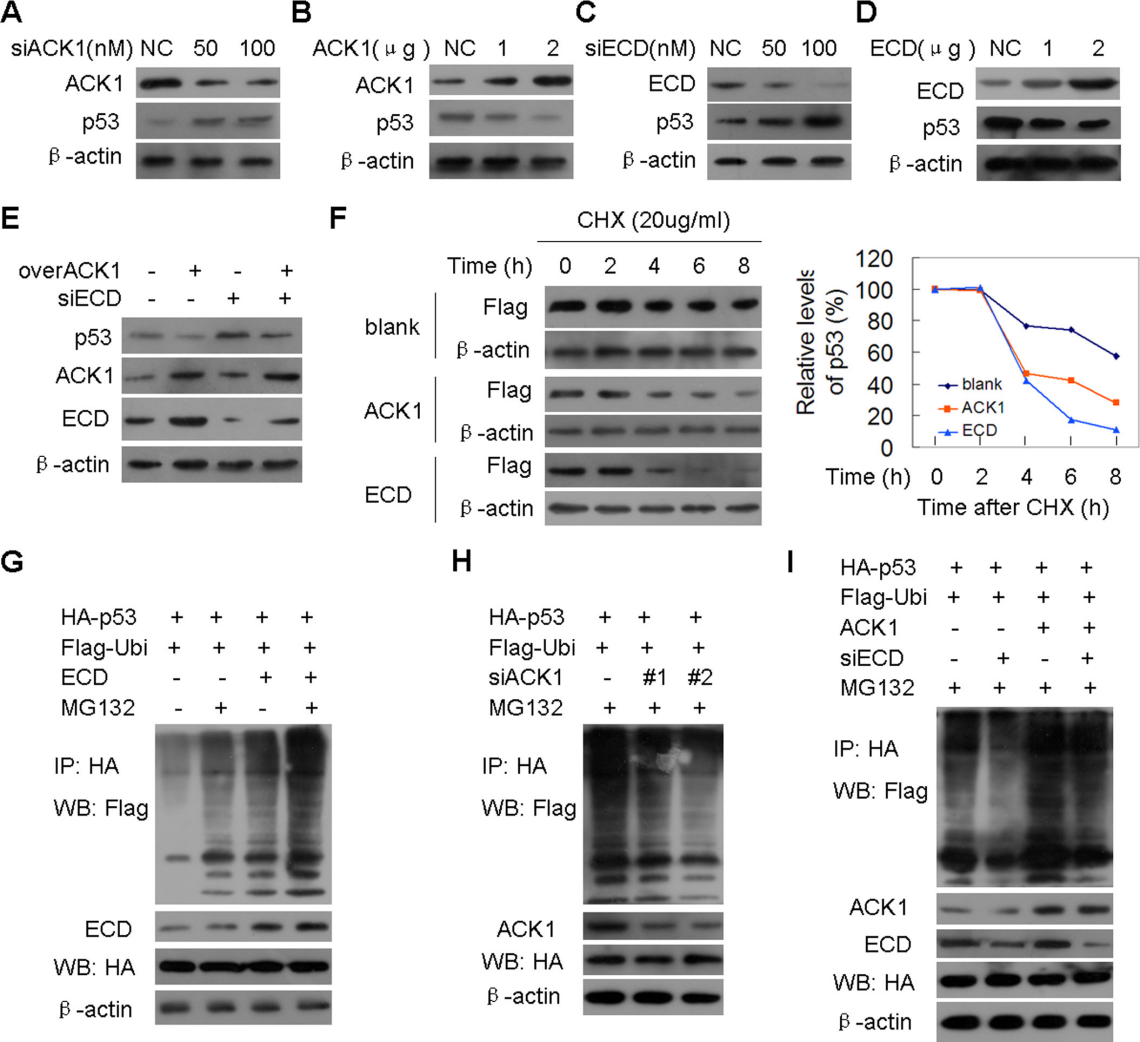
ACK1 decreases tumor suppressor p53 levels via ECD-dependent p53 protein ubiquitination degradation **(A, C)** SGC-7901 cells were transfected with the indicated concentrations of anti-ACK1 **(A)** or anti-ECD **(B)** siRNA, and p53 levels were detected by western blot. (B, D) SGC-7901 cells were transfected with the indicated amount of ACK1 **(B)** or ECD **(D)** plasmid, and p53 levels were determined by western blot. **(E)** SGC-7901 cells were co-transfected with the ACK1 plasmid together with an anti-ECD siRNA, and p53 levels were detected by western blot. **(F)** SGC-7901 cells were transfected with ACK1 or ECD plasmid prior to exposure to the protein synthesis inhibitor cycloheximide (CHX) for the indicated time. The p53 protein levels were analyzed by western blot (left panel), and the densities of the p53 protein bands at each time point were normalized to β-actin and were calculated into percentages using 100% as the value of the zero time point (right panel). (G-I) SGC-7901 cells were co-transfected with the indicated plasmids or siRNAs for 36h and were then treated with or without 20 μg/ml MG132 for 2 h. The cell lysates were resolved directly by SDS-PAGE or were first immunoprecipitated with HA antibody and the indicated proteins were then detected by western blot.

A previous study demonstrated that ECD interacts with MDM2 and up-regulates p53 by inhibiting the MDM2-mediated degradation of p53 [24]. Additionally, p53 plays an important role in controlling the entry into mitosis or the induction of apoptosis when cells enter G2 with damaged DNA, in addition its important role in regulating the G1/S transition [25, 26]. Therefore, we sought to determine whether ACK1 down-regulates p53 expression in an ECD-dependent manner. We found that silencing of ACK1 increased p53 protein levels in a dose-dependent manner, while ACK1 over-expression decreased p53 levels (Figure 7A and 7B). ECD is as an important downstream effector of ACK1 in the regulation of tumor growth and colony formation. We further demonstrated that silencing of ECD increased p53 levels in a dose-dependent manner, while ECD over-expression decreased p53 levels, similar to the effects of ACK1 (Figure 7C and 7D). Silencing of ECD attenuated the down-regulation of p53 induced by ACK1 over-expression (Figure 7E). Moreover, a negative correlation was observed between ACK1/ECD and p53, whereas positive correlation was observed between ACK1 and ECD in the xenograft tumor tissues (Supplementary Figure S3).

To investigate the regulatory mechnism of ACK1 and ECD on p53, the half-life and ubiquitination level of p53 were determined. We found that the half-life of the p53 protein was consistently shorter when ECD or ACK1 was over-expressed (Figure 7F), suggesting that p53 degradation was induced by ACK1 and ECD. The p53 protein was more heavily ubiquitied in the presence of the ectopically expressed ECD or ACK1 and MG132 treatment (Figure 7G, lane 1 vs lane 3 in Fig 7I), while silencing of ACK1 or ECD decreased the ubiquitination level of p53 protein (Figure 7H, lane 1 and 2 in Figure 7I). Silencing of ECD blocked the ubiquitination enhancement of p53 protein induced by ACK1 over-expression (lane 3 vs lane 4 in Figure 7I). Taken together, ACK1 amplification decreased p53 protein level through ECD-dependent p53 ubiquitination degradation.

## DISCUSSION

In this study, we demonstrate that the ACK1 gene is amplified in GC and that silencing of ACK1 induces GC cell G2/M arrest and apoptosis and inhibits tumor growth and colony formation by inducing an ECD-mediated p53 increase in GC cells. Chemotherapy is the mainstay treatment for locally advanced or metastatic GC patients [27]. Although great efforts have been made to improve the prevention and treatment of GC patients through chemotherapy, the 5-year survival of GC patients remains low [28]. Targeted small molecules or antibody therapies designed to inhibit a specific addictive oncogene are a promising therapeutic strategy. Our data indicate that silencing of ACK1 significantly inhibited GC cell proliferation and colony formation and induced G2/M arrest and apoptosis *in vitro*, suppressed tumor growth *in vivo*, indicating that ACK1 may be a novel drug target for GC.

ACK1, is a non-receptor tyrosine kinase that is aberrantly activated, amplified or mutated in breast, prostate and non-small-cell lung cancer and hepatocellular carcinoma [5, 6, 8, 10–12, 15]. Since the first clinical success of the tyrosine kinase inhibitors Imatinib and Gleevec against Bcr-Abl in chronic myeloid leukemia, many tyrosine kinase inhibitors have been clinically used. Selective targeting of HER2 with Trastuzumab in HER2^+^ breast cancers and mutant B-Raf^V600E^ with Vemurafenib (PLX-4032) in melanoma has exhibited effective anti-cancer effects [5]. Due to the oncogenic role of ACK1 in the breast and prostate, great efforts are being made by multiple groups towards developing highly potent and specific small molecule inhibitors targeting ACK1 in breast and prostate cancers. At least ten small molecule inhibitors against ACK1 have presently been reported in the literature [5, 29]. If these inhibitors will be available in clinical trials, a subset of GC patients with ACK1 amplification will likely benefit from therapy with these ACK1 potential inhibitors.

ACK1 not only activates survival kinase AKT and up-regulates the activity of hormone receptor androgen receptor but also induces tumor suppressor WWOX degradation in hormone-driven breast and prostate cancers [15, 30]. Our previous study showed that ACK1 promotes gastric cancer EMT and metastasis by AKT1-POU2F1-ECD signaling axis, in which ACK1 induces phosphorylation of AKT at Thr 308 and Ser 473 to increase the expression of the transcription factor POU2F1, which regulates the expression of the novel protein ECD by binding to the ECD promoter [16]. How does ACK1 amplification promote gastric tumorigenesis through ECD pathway? In this study, a novel molecular mechanism for ACK1 in cancer progression was elucidated in GC, a non-hormone-driven cancer, whereby ACK1 promotes tumor growth, survival and colony formation through an ECD-dependent p53 ubiquitination degradation. As p53 is a key classic tumor suppressor gene and cell cycle regulator, tumorigenesis can be inhibited by p53-mediated cell cycle arrest, apoptotic and/or cellular senescence [31]. Additionally p53 plays an important role in controlling entry into mitosis or inducing apoptosis when DNA damaged cells enter G2 [25]. Here, we demonstrate that silencing of ACK1 or ECD induced G2/M arrest and apoptosis in GC cells and down-regulated p53 ubiquitination level and up-regulated tumor suppressor p53 level. These observations indicate that over-expressed ACK1 promotes gastric tumorigenesis via the down-regulation of p53.

A previous study showed that ECD interacts with MDM2 and up-regulates p53 by inhibiting MDM2-mediated degradation of p53 in osteosarcoma [24]. ECD over-expression increases p53 target gene transcription and leads to p53-dependent accelerated cellular senescence, whereas transient knockdown of ECD increases cell proliferation in p53-positive but not p53-negative cells [24], suggesting that ECD is a tumor suppressor. However, our data here show that silencing of ECD induces cell cycle arrest and apoptosis and inhibits GC cell colony formation. Over-expression of ECD down-regulates p53 tumor suppressor levels, and silencing of ECD increases p53 levels. Our data indicate that ECD is an oncogene in GC. This finding is consistent with previous reports. In Drosophila, ECD mutants exhibit a general defect in cell survival [32]; in yeast, ECD can rescue the growth defect of the GCR2 mutation [21]. The conditional deletion of ECD in murine embryonic fibroblast cells leads to G1/S arrest [22]. Additionally, knockdown of ECD reduces pancreatic cancer cell growth and tumorigenicity [23]. Together, these observations indicate that the ECD oncogene promotes cell proliferation, cell survival and tumor growth by inducing p53 ubiquitination and decreasing the expression of the tumor suppressor p53.

In summary, the ACK1 gene is amplified in GC, and silencing of ACK1 induces G2/M arrest and apoptosis, inhibits GC cell proliferation, colony formation and tumor growth. Additionally, we revealed a novel molecular mechanism of ACK1 amplification in gastric tumorigenesis by which ACK1 promotes the tumor suppressor p53 protein ubiquitination degradation and down-regulates p53 levels in an ECD-dependent manner. ACK1 may serve as a novel therapeutic target for GC.

## MATERIALS AND METHODS

### Cell culture and RNAi

SGC-7901 and MGC-803 gastric cancer cells were from the National Infrastructure of Cell Line Resources of China and were cultured in RPMI 1640 medium supplemented with 10% fetal bovine serum (FBS) plus antibiotics at 37°C in a 5% CO_2_ atmosphere. SiRNAs against ACK1 and ECD genes and corresponding scrambled siRNAs (GenePharma) were transfected into SGC-7901 cells with RNAiMAX (Invitrogen) for 48h (unless otherwise stated). The following siRNA sequences were used: siACK1#1, sense: 5′-CUCAGAAUGACGACCAUUATT-3′, anti-sense: 5′-UAAUGGUCGUCAUUCUGAGTT-3′; siACK1#2, sense: 5′-CCCACUUUGAGUACGUCAATT-3′, anti-sense: 5′-UUGACGUACUCAAAGUGGGTT-3′; siACK1#3, sense: 5′-GCACCCACUAUUACUUGCUTT-3′; anti-sense: 5′-AGCAAGUAAUAGUGGGUGCTT-3′. The anti-ECD siRNA and negative control siRNA sequences were as previously reported [16].

### Semi-quantitative RT-PCR and real-time RT-PCR assay

Total RNA was extracted from cells using Trizol total RNA isolation reagent (Invitrogen). RNA reverse transcription was performed by using the Reverse Transcriptase M-MLV (Invitrogen), cDNA was synthesized from total RNA using random primer oligo(dT)18 (Takara). Quantitative RT-PCR was carried out using SYBR Premix Ex Taq (Takara). The following primers were used: ACK1, forward: 5′-AGAGCCTGAAGACACGCACC-3′, reverse: 5′-GGATCTGACTGCCGTTGAGG-3′; GAPDH (internal control), forward: 5′- GAAGGTGAAGGTCGGAGTC -3′, reverse: 5′- AAGATGGTGATGGGATTTC -3′.

The average of three independent analyses was calculated.

### Western blot

Cellular proteins were prepared as previously described [33]. In brief, protein extracts were separated by 10% SDS-PAGE and then electroblotted onto a polyvinylidene fluoride membrane, which was then incubated with the indicated primary antibodies at 4°C overnight, respectively, followed by incubation with corresponding secondary antibodies at room temperature. The primary antibodies here used were as follows: ACK1 (ab135672, Abcam), ECD (10192–1–AP, Proteintech), cyclinB1 (sc-752, Santa Cruz), Cdc2 (sc-747, Santa Cruz), Pro-caspase 3 (sc-7148, Santa Cruz), PARP (#9532, CST), p53 (sc-126, Santa Cruz) and β-actin (sc-81178, Santa Cruz).

### Construct of cell lines with stable silencing of ACK1

The SGC-7901-LUC-ACK1 shRNA cell line with stable silencing of ACK1 and corresponding negative control cell line SGC-7901-LUC-NC, the SGC-7901-LUC-ECD shRNA cell line with stable silencing of ECD and corresponding negative control cell line SGC-7901-LUC-NC, were previously constructed [16].

### Colony formation assays

Two hundred and fifty cells were plated in 6-well culture plates and cultured for two weeks. These cells were then fixed with methanol and stained with crystal violet solution. The numbers of colonies containing ≥ 30 cells were counted under the microscope. These experiments were repeated three times.

### Cell cycle analysis

The cells were washed twice with ice-cold PBS, fixed with 75% ethanol and treated with RNase at 37°C for 1 h, and they were then stained with Propidium Iodide (PI) for 30 min in dark. The DNA content of each cell was measured with a flow cytometer (FACS Calibur, Becton Dickinson, MD, USA). The proportion of DNA was analyzed using Cell Quest and Modfit LT version 3.0 Software.

### Apoptosis assay

The SGC-7901 and MGC-803 cells were transfected with anti-ACK1 siRNA#1, anti-ACK1 siRNA#2, anti- ECD siRNA#1 or anti-ECD siRNA#2, or they were co-transfected with anti-ACK1 siRNA together with anti-ECD siRNA. These cells were harvested and incubated with AnnexinV-FITC and PI for 10 min in the dark. These samples were then analyzed at 525 nm for FITC and at 630 nm for PI using a FACStar Plus flow cytometer.

### *In vivo* tumor growth assay

Male BALB/c nude mice (4–5 weeks old) were purchased from the Laboratory Animal Center, Sun Yat-sen University (Guangzhou, China) and were bred and maintained under defined conditions at the Animal Experiment Center of College of Medicine (SPF grade), Jinan University. Animal experiments were approved by the Laboratory Animal Ethics Committee of Jinan University and conformed to the legal mandates and national guidelines for the care and maintenance of laboratory animals. The *in vivo* tumor growth assay was performed as previously described [34]. In brief, 2 × 10^6^ SGC-7901-LUC-ACK1 shRNA or SGC-7901-LUC-NC cells was subcutaneously injected into the dorsal flanks of each mouse (*n* = 5). Or 2 × 10^6^ SGC-7901 cells were subcutaneously injected into the dorsal flanks of each mouse. After one week of injection, 10 nmol cholesterol-conjugated siACK1 in 0.1 ml saline buffer were locally injected into the tumor mass once every three days due to the absence of commercially available ACK1-specific small molecule inhibitor. After 3 weeks, the mice were euthanized, and the tumors were dissected and weighed.

### Gene ontology (GO) enrichment analysis

The 147 differential proteins regulated by ACK1 in previous report [16] were further analyzed by GO annotation as previous described [2, 35]. Gene Ontology (GO) enrichment analysis was implemented by Bioconductor package GeneAnswers [36]. The significant GO categories were filtered by the criteria of *p*-value with FDR < 0.01.

### Statistics

The statistical analysis was performed using the statistical software package Prism 5. Differences between the two groups were compared using Student's *t*-test. *p < 0.05 or **p < 0.001 was considered to be significant.

## SUPPLEMENTARY FIGURES



## Abbreviations

ACK1: activated Cdc42-associated kinase 1
ECD: ecdysoneless homolog
GC: gastric cancer
EMT: epithelial-mesenchymal transition

## References

[1] Jemal A, Bray F, Center MM, Ferlay J, Ward E, Forman D (2011). Global cancer statistics. CA Cancer J Clin.

[2] Yan GR, Xu SH, Tan ZL, Yin XF, He QY (2011). Proteomics characterization of gastrokine 1-induced growth inhibition of gastric cancer cells. Proteomics.

[3] Riquelme I, Saavedra K, Espinoza JA, Weber H, Garcia P, Nervi B, Garrido M, Corvalan AH, Roa JC, Bizama C (2015). Molecular classification of gastric cancer: Towards a pathway-driven targeted therapy. Oncotarget.

[4] Liang L, Fang JY, Xu J (2015). Gastric cancer and gene copy number variation: emerging cancer drivers for targeted therapy. Oncogene.

[5] Mahajan K, Mahajan NP (2013). ACK1 tyrosine kinase: targeted inhibition to block cancer cell proliferation. Cancer Lett.

[6] van der Horst EH, Degenhardt YY, Strelow A, Slavin A, Chinn L, Orf J, Rong M, Li S, See LH, Nguyen KQ, Hoey T, Wesche H, Powers S (2005). Metastatic properties and genomic amplification of the tyrosine kinase gene ACK1. Proc Natl Acad Sci U S A.

[7] Shinmura K, Kiyose S, Nagura K, Igarashi H, Inoue Y, Nakamura S, Maeda M, Baba M, Konno H, Sugimura H (2014). TNK2 gene amplification is a novel predictor of a poor prognosis in patients with gastric cancer. J Surg Oncol.

[8] Mendez P, Ramirez JL (2013). Copy number gains of FGFR1 and 3q chromosome in squamous cell carcinoma of the lung. Transl Lung Cancer Res.

[9] Manser E, Leung T, Salihuddin H, Tan L, Lim L (1993). A non-receptor tyrosine kinase that inhibits the GTPase activity of p21cdc42. Nature.

[10] Hu F, Liu H, Xie X, Mei J, Wang M (2015). Activated cdc42-associated kinase is up-regulated in non-small-cell lung cancer and necessary for FGFR-mediated AKT activation. Mol Carcinog.

[11] Mahajan K, Mahajan NP (2015). ACK1/TNK2 tyrosine kinase: molecular signaling and evolving role in cancers. Oncogene.

[12] Mahajan K, Lawrence HR, Lawrence NJ, Mahajan NP (2014). ACK1 tyrosine kinase interacts with histone demethylase KDM3A to regulate the mammary tumor oncogene HOXA1. J Biol Chem.

[13] La Torre A, del Mar Masdeu M, Cotrufo T, Moubarak RS, del Rio JA, Comella JX, Soriano E, Urena JM (2013). A role for the tyrosine kinase ACK1 in neurotrophin signaling and neuronal extension and branching. Cell Death Dis.

[14] Mahajan K, Coppola D, Chen YA, Zhu W, Lawrence HR, Lawrence NJ, Mahajan NP (2012). Ack1 tyrosine kinase activation correlates with pancreatic cancer progression. Am J Pathol.

[15] Mahajan K, Mahajan NP (2010). Shepherding AKT and androgen receptor by Ack1 tyrosine kinase. J Cell Physiol.

[16] Xu SH, Huang JZ, Xu ML, Yu G, Yin XF, Chen D, Yan GR (2015). ACK1 promotes gastric cancer epithelial-mesenchymal transition and metastasis through AKT-POU2F1-ECD signalling. J Pathol.

[17] Kiyose S, Nagura K, Tao H, Igarashi H, Yamada H, Goto M, Maeda M, Kurabe N, Suzuki M, Tsuboi M, Kahyo T, Shinmura K, Hattori N, Sugimura H (2012). Detection of kinase amplifications in gastric cancer archives using fluorescence in situ hybridization. Pathol Int.

[18] Deng N, Goh LK, Wang H, Das K, Tao J, Tan IB, Zhang S, Lee M, Wu J, Lim KH, Lei Z, Goh G, Lim QY, Tan AL, Sin Poh DY, Riahi S (2012). A comprehensive survey of genomic alterations in gastric cancer reveals systematic patterns of molecular exclusivity and co-occurrence among distinct therapeutic targets. Gut.

[19] Chen X, Leung SY, Yuen ST, Chu KM, Ji J, Li R, Chan AS, Law S, Troyanskaya OG, Wong J, So S, Botstein D, Brown PO (2003). Variation in gene expression patterns in human gastric cancers. Mol Biol Cell.

[20] D'Errico M, de Rinaldis E, Blasi MF, Viti V, Falchetti M, Calcagnile A, Sera F, Saieva C, Ottini L, Palli D, Palombo F, Giuliani A, Dogliotti E (2009). Genome-wide expression profile of sporadic gastric cancers with microsatellite instability. Eur J Cancer.

[21] Sato T, Jigami Y, Suzuki T, Uemura H (1999). A human gene, hSGT1, can substitute for GCR2, which encodes a general regulatory factor of glycolytic gene expression in Saccharomyces cerevisiae. Mol Gen Genet.

[22] Kim JH, Gurumurthy CB, Naramura M, Zhang Y, Dudley AT, Doglio L, Band H, Band V (2009). Role of mammalian Ecdysoneless in cell cycle regulation. J Biol Chem.

[23] Dey P, Rachagani S, Chakraborty S, Singh PK, Zhao X, Gurumurthy CB, Anderson JM, Lele S, Hollingsworth MA, Band V, Batra SK (2012). Overexpression of ecdysoneless in pancreatic cancer and its role in oncogenesis by regulating glycolysis. Clin Cancer Res.

[24] Zhang Y, Chen J, Gurumurthy CB, Kim J, Bhat I, Gao Q, Dimri G, Lee SW, Band H, Band V (2006). The human orthologue of Drosophila ecdysoneless protein interacts with p53 and regulates its function. Cancer Res.

[25] Taylor WR, Stark GR (2001). Regulation of the G2/M transition by p53. Oncogene.

[26] Deben C, Wouters A, Op de Beeck K, van Den Bossche J, Jacobs J, Zwaenepoel K, Peeters M, Van Meerbeeck J, Lardon F, Rolfo C, Deschoolmeester V, Pauwels P (2015). The MDM2-inhibitor Nutlin-3 synergizes with cisplatin to induce p53 dependent tumor cell apoptosis in non-small cell lung cancer. Oncotarget.

[27] Wagner AD, Grothe W, Haerting J, Kleber G, Grothey A, Fleig WE (2006). Chemotherapy in advanced gastric cancer: a systematic review and meta-analysis based on aggregate data. J Clin Oncol.

[28] Power DG, Kelsen DP, Shah MA (2010). Advanced gastric cancer–slow but steady progress. Cancer Treat Rev.

[29] Lawrence HR, Mahajan K, Luo Y, Zhang D, Tindall N, Huseyin M, Gevariya H, Kazi S, Ozcan S, Mahajan NP, Lawrence NJ (2015). Development of novel ACK1/TNK2 inhibitors using a fragment-based approach. J Med Chem.

[30] Mahajan NP, Whang YE, Mohler JL, Earp HS (2005). Activated tyrosine kinase Ack1 promotes prostate tumorigenesis: role of Ack1 in polyubiquitination of tumor suppressor Wwox. Cancer Res.

[31] Li T, Kon N, Jiang L, Tan M, Ludwig T, Zhao Y, Baer R, Gu W (2012). Tumor suppression in the absence of p53-mediated cell-cycle arrest, apoptosis, and senescence. Cell.

[32] Gaziova I, Bonnette PC, Henrich VC, Jindra M (2004). Cell-autonomous roles of the ecdysoneless gene in Drosophila development and oogenesis. Development.

[33] Chen D, Dang BL, Huang JZ, Chen M, Wu D, Xu ML, Li R, Yan GR (2015). MiR-373 drives the epithelial-to-mesenchymal transition and metastasis via the miR-373-TXNIP-HIF1alpha-TWIST signaling axis in breast cancer. Oncotarget.

[34] Tang J, Wang G, Zhang M, Li FY, Sang Y, Wang B, Hu K, Wu Y, Luo R, Liao D, Cao J, Wang X, Wang L, Zhang R, Zhang X, Deng WG (2014). Paradoxical role of CBX8 in proliferation and metastasis of colorectal cancer. Oncotarget.

[35] Yan GR, Zou FY, Dang BL, Zhang Y, Yu G, Liu X, He QY (2012). Genistein-induced mitotic arrest of gastric cancer cells by downregulating KIF20A, a proteomics study. Proteomics.

[36] Yu G, Wang LG, Han Y, He QY (2012). clusterProfiler: an R package for comparing biological themes among gene clusters. OMICS.

