# Supplementary material for: Amplification of ACK1 promotes gastric tumorigenesis via ECD-dependent p53 ubiquitination degradation
Source: Oncotarget. 2015 Oct 20;8(8):12705–16. doi: 10.18632/oncotarget.6194 (PMC5355047; doi:10.18632/oncotarget.6194)
Supplement: Supplementary file 1 [file oncotarget-08-12705-s001.pdf]

Amplification of ACK1 promotes gastric tumorigenesis via ECD-dependent p53 ubiquitination degradation

Supplementary Materials

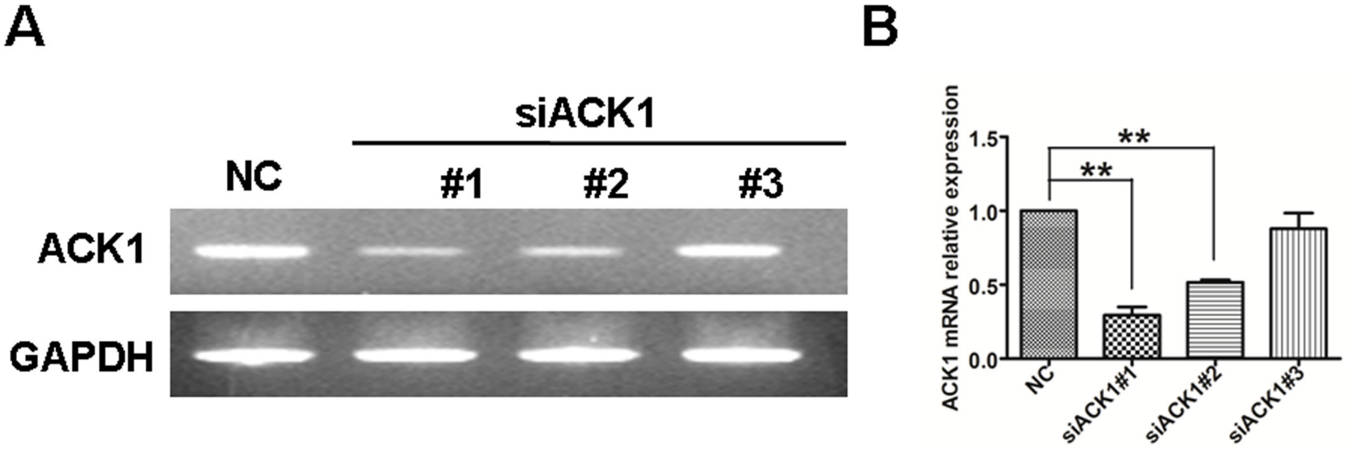

Supplementary Figure S1. ACK1 was silenced by anti-ACK1 siRNAs. SGC-7901 cells were transfected with the indicated anti-ACK1 siRNAs for 48 h. ACK1 mRNA levels were determined by RT-PCR and qRT-PCR.

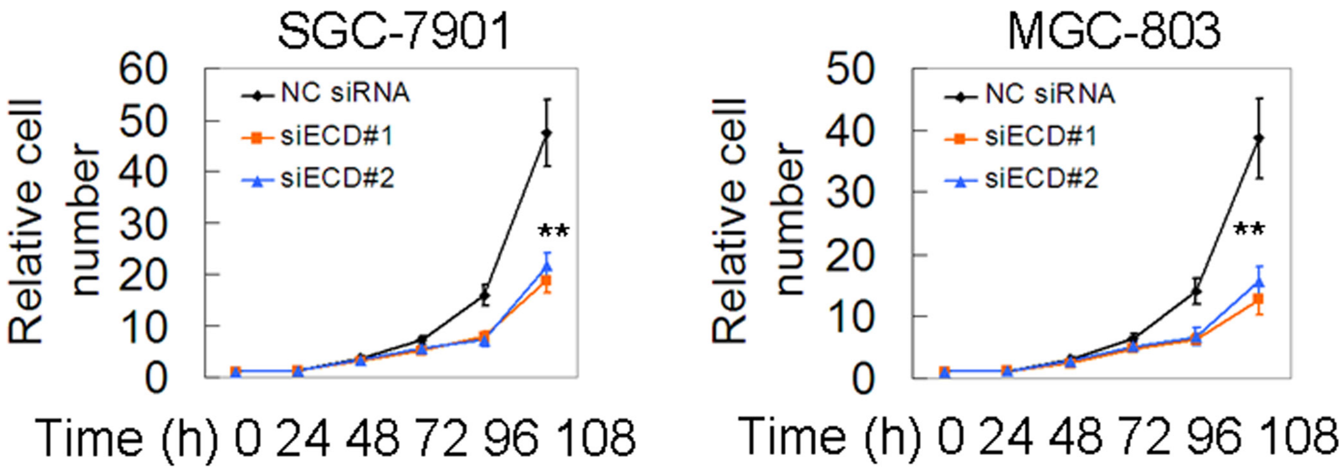

Supplementary Figure S2. Silencing of ECD inhibited GC cell proliferation. SGC-7901 and MGC-803 cells were transfected with the indicated siRNAs for the indicated time, and the cell number was counted.

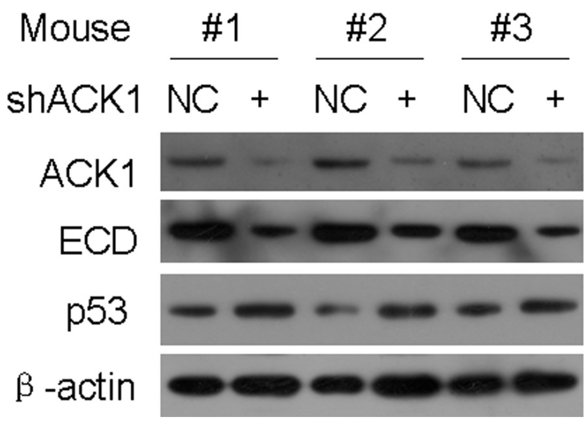

Supplementary Figure S3. A negative correlation was observed between ACK1/ECD and p53, whereas positive correlation was observed between ACK1 and ECD in the xenograft tumor tissues. The indicated proteins in the xenograft tumor tissues were detected by western blot.
